# Extragenital Chlamydia and Gonorrhea Among Community Venue–Attending Men Who Have Sex with Men — Five Cities, United States, 2017

**DOI:** 10.15585/mmwr.mm6814a1

**Published:** 2019-04-12

**Authors:** Michelle L. Johnson Jones, Johanna Chapin-Bardales, Destani Bizune, John R. Papp, Christi Phillips, Robert D. Kirkcaldy, Cyprian Wejnert, Kyle T. Bernstein, National HIV Behavioral Surveillance Sexually Transmitted Infection Study Group, National HIV Behavioral Surveillance Sexually Transmitted Infection Study Group, Salma Khuwaja, Zaida Lopez, Paige Padgett, David Forrest, Willie Nixon, Emma Spencer, Sarah Braunstein, Sidney Carrillo, Alexis Rivera, Theresa Ick, H. Fisher Raymond, Jenevieve Opoku, Irene Kuo

**Affiliations:** ^1^Division of STD Prevention, National Center for HIV/AIDS, Viral Hepatitis, STD, and TB, CDC; ^2^Division of HIV/AIDS Prevention, National Center for HIV/AIDS, Viral Hepatitis, STD, and TB, CDC; ^3^Oak Ridge Institute for Science and Education, Oak Ridge, Tennessee.; Houston Health Department; Houston Health Department; University of Texas Health Science Center; University of Miami; Florida Department of Health; Florida Department of Health; New York City Department of Health and Mental Hygiene; New York City Department of Health and Mental Hygiene; New York City Department of Health and Mental Hygiene; San Francisco Department of Public Health; San Francisco Department of Public Health; District of Columbia Department of Health; George Washington University

## Abstract

Sexually transmitted diseases (STDs) disproportionately affect gay, bisexual, and other men who have sex with men (MSM) in the United States (*1*). Because chlamydia and gonorrhea at extragenital (rectal and pharyngeal) anatomic sites are often asymptomatic, these anatomic sites serve as a reservoir of infection, which might contribute to gonococcal antimicrobial resistance (*2*) and increased risk for human immunodeficiency virus (HIV) transmission and acquisition (*3*). To ascertain prevalence of extragenital STDs, MSM attending community venues were recruited in five U.S. cities to provide self-collected swabs for chlamydia and gonorrhea screening as part of National HIV Behavioral Surveillance (NHBS). Overall, 2,075 MSM provided specimens with valid results, and 13.3% of participants were infected with at least one of the two pathogens in at least one of these two extragenital anatomic sites. Approximately one third of participating MSM had not been screened for STDs in the previous 12 months. MSM attending community venues had a high prevalence of asymptomatic extragenital STDs. The findings underscore the importance of sexually active MSM following current recommendations for STD screening at all exposed anatomic sites at least annually (*4*).

According to a systematic review of studies from 2000 to 2016, the estimated prevalences of rectal chlamydia and gonorrhea among MSM were 9.0% and 6.1%, respectively (*5*). Fewer data are available on pharyngeal chlamydia and gonorrhea; prevalence estimates were 0%–3.6% for pharyngeal chlamydia and 0%–16.5% for pharyngeal gonorrhea among MSM (*6*). Nearly all reported prevalences of extragenital infections among MSM have been estimated from clinic-based samples of patients. Because men in these samples sought clinical care (and could be at elevated risk for STDs, especially if seen at an STD clinic), reported estimates might not reflect prevalences among a broader population of MSM. To inform the epidemiology of bacterial STDs among MSM, extragenital chlamydia and gonorrhea screening was offered to MSM recruited to participate in NHBS at MSM-frequented venues in five U.S. cities (Houston, Texas; Miami, Florida; New York City, New York; San Francisco, California; and Washington, DC). NHBS assessed adherence to current screening recommendations using the question “In the past 12 months, were you tested by a doctor or other health care provider for a sexually transmitted disease like gonorrhea, chlamydia, or syphilis? Do not include tests for HIV or hepatitis.”

NHBS conducts anonymous behavioral surveys on a rotating basis among populations with elevated HIV risk in the United States (*7*). In 2017, MSM participants were recruited from MSM-frequented community venues (e.g., bars, clubs, fitness centers, and other locations patronized by MSM) and were eligible if they were male at birth, identified as male, were aged ≥18 years, reported ever having sex with a male, were residing in the city of administration, had not previously completed the NHBS survey in the current cycle, and could complete the survey in English or Spanish. This analysis was restricted to participants who had sex with a male in the previous 12 months. Participants completed an interviewer-administered standardized computer-assisted personal interview survey that collected sociodemographic and epidemiologic characteristics. All participants were offered an anonymous HIV test. Monetary tokens of appreciation for participating were provided to participants; amounts were determined locally. NHBS activities were reviewed at CDC as nonengaged research and approved by local institutional review boards for each participating location.

NHBS participants were offered additional tokens of appreciation for providing anonymous self-collected rectal and pharyngeal swabs for chlamydia and gonorrhea testing. CDC tested specimens from four of the cities using the Aptima Combo 2 Panther system (Hologic), and the San Francisco Department of Public Health Laboratory tested specimens from San Francisco using the same assay. Test results were communicated back to local NHBS teams for notification and treatment referrals when indicated, using numeric identifiers to maintain participants’ anonymity. Test results were linked with completed survey data and HIV test results. STD prevalence was calculated as the number of persons with positive test results divided by the total number of persons tested with a valid result, stratified by anatomic site (rectum and oropharynx) and STD (chlamydia and gonorrhea) with 95% Wald confidence intervals (CIs) and bivariate analyses for comparing characteristics. Analyses were performed using SAS software (version 9.4; SAS Institute).

Among 2,371 eligible MSM who participated in NHBS in the five cities, 2,077 (87.6%) provided specimens for STD testing, 2,044 (98.4%) of whom provided both rectal and pharyngeal swabs. Analysis included 2,075 participants, after excluding two who lacked valid results. Overall, 13.3% (95% CI = 11.8%–14.8%) of participants were infected with at least one of the two STDs at one or two anatomic sites. Prevalence of rectal chlamydia (7.3%) was higher than that of rectal gonorrhea (4.5%; p<0.001), whereas prevalence of pharyngeal gonorrhea (4.6%) was higher than that of pharyngeal chlamydia (1.4%; p<0.001) (Figure). Rectal gonorrhea prevalence was higher among MSM who reported being HIV-positive than among those who were HIV-negative (8.2% versus 3.3%; p<0.001) (Table). Prevalences of both pharyngeal infections were similar among those testing HIV-positive and HIV-negative. Prevalence of infection was higher in younger men (aged 18–29 years), compared with older men for each type and anatomic site of infection except pharyngeal chlamydia. Black and Hispanic MSM had higher prevalences of pharyngeal gonorrhea than did white MSM, otherwise, no differences were observed by racial/ethnic categories. San Francisco had the lowest prevalences for each pathogen and anatomic site; prevalences for each infection varied by city of residence (Table).

**FIGURE:**
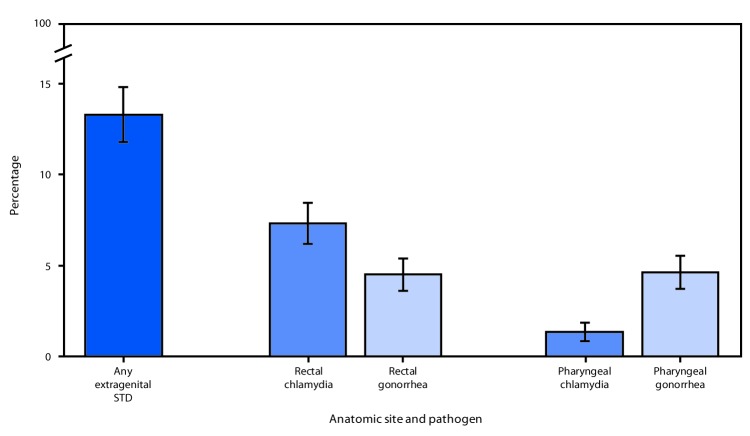
Prevalence of extragenital chlamydia and gonorrhea among community venue–attending* men who have sex with men, by anatomic site — National HIV Behavioral Surveillance, five U.S. cities^†^, 2017 **Abbreviations: **HIV = human immunodeficiency virus; STD = sexually transmitted disease. * Community venues include bars, clubs, fitness centers, and other locations frequented by men who have sex with men. ^†^ Houston, Texas; Miami, Florida; New York City, New York; San Francisco, California; Washington, DC.

**TABLE:** Characteristics of participants and prevalence of extragenital chlamydia and gonorrhea among community venue–attending* men who have sex with men, by anatomic site — National HIV Behavioral Surveillance, five U.S. cities, 2017

Characteristic	No. (%) of all participants	Prevalence (95% CI)			
		Rectal chlamydia (no. tested = 2,024)	Rectal gonorrhea (no. tested = 2,023)	Pharyngeal chlamydia (no. tested = 2,072)	Pharyngeal gonorrhea (no. tested = 2,072)
**Overall**	**2,075 (100)**	**7.3 (6.2–8.5)**	**4.5 (3.6–5.4)**	**1.4 (0.9–1.9)**	**4.6 (3.7–5.5)**
**Age group (yrs)**					
18–29	737 (35.5)	9.2 (7.1–11.4)	6.2 (4.5–8.0)	1.8 (0.8–2.7)	6.0 (4.3–7.7)
30–39	676 (32.6)	7.4 (5.4–9.4)	4.2 (2.7–5.8)	1.3 (0.5–2.2)	4.9 (3.3–6.5)
40–49	330 (15.9)	6.2 (3.6–8.8)	3.4 (1.4–5.4)	0.9 (0.0–1.9)	3.3 (1.4–5.3)
≥50	332 (16.0)	3.8 (1.7–5.9)	2.2 (0.6–3.9)	0.9 (0.0–1.9)	2.4 (0.8–4.1)
**Race/Ethnicity**					
Hispanic	733 (35.3)	7.5 (5.6–9.5)	4.9 (3.3–6.5)	2.0 (1.0–3.1)	5.3 (3.7–7.0)
White, non-Hispanic	688 (33.2)	7.3 (5.3–9.3)	3.9 (2.4–5.3)	0.9 (0.2–1.6)	3.2 (1.9–4.5)
Black, non-Hispanic	455 (21.9)	7.2 (4.8–9.6)	5.6 (3.5–7.7)	1.3 (0.3–2.4)	6.6 (4.3–8.9)
Other	187 (9.0)	7.3 (3.5–11.1)	2.2 (0.1–4.4)	0.5 (0.0–1.6)	2.7 (0.4–5.0)
Unknown	12 (0.6)	N/A	N/A	N/A	N/A
**HIV test results^†^**					
HIV-negative	1,577 (76.0)	6.6 (5.3–7.8)	3.3 (2.4–4.1)	1.3 (0.7–1.8)	4.3 (3.3–5.3)
HIV-positive					
Self-reported HIV-positive	386 (18.6)	9.0 (6.1–11.9)	8.2 (5.5–11.0)	1.6 (0.3–2.8)	5.2 (3.0–7.4)
Did not self-report HIV-positive	73 (3.5)	9.9 (2.9–16.8)	5.6 (0.3–11.0)	2.7 (0.0–6.5)	5.5 (0.3–10.7)
No valid test results available	39 (1.9)	N/A	N/A	N/A	N/A
**STD testing in previous 12 months^§^**					
Tested	1,371 (66.1)	7.1 (5.7–8.5)	4.2 (3.1–5.3)	1.2 (0.6–1.7)	4.5 (3.4–5.6)
Not tested	698 (33.6)	7.8 (5.8–9.8)	5.0 (3.4–6.6)	1.7 (0.8–2.7)	4.7 (3.2–6.3)
Don't know/Skipped	6 (0.3)	N/A	N/A	N/A	N/A
**City of residence**					
Houston, Texas	468 (22.6)	8.0 (5.5–10.4)	6.7 (4.4–9.0)	2.8 (1.3–4.3)	6.2 (4.0–8.4)
Miami, Florida	345 (16.6)	5.6 (3.1–8.0)	5.6 (3.1–8.0)	1.4 (0.2–2.7)	4.6 (2.4–6.9)
New York City, New York	425 (20.5)	7.2 (4.7–9.7)	4.3 (2.4–6.3)	0.9 (0.0–1.9)	3.8 (2.0–5.6)
San Francisco, California	418 (20.1)	5.2 (3.0–7.4)	1.8 (0.5–3.1)	0.7 (0.0–1.5)	3.6 (1.8–5.4)
Washington, DC	419 (20.2)	10.1 (7.2–13.1)	3.9 (2.0–5.7)	0.7 (0.0–1.5)	4.8 (2.7–6.8)

**Abbreviations:** CI = confidence interval; HIV = human immunodeficiency virus; N/A = not applicable; STD = sexually transmitted disease.

* Community venues include bars, clubs, fitness centers, and other locations frequented by men who have sex with men.

^†^ Based on results of rapid HIV laboratory testing conducted during National HIV Behavior Surveillance encounter.

^§^ Self-reported.

Overall, 698 (33.6%) MSM participants reported that they had not been tested for an STD in the previous 12 months (Table). Prevalence was similar for MSM who did and did not report recent STD testing, irrespective of anatomic site or pathogen.

## Discussion

In a community venue–based sample of sexually active MSM, approximately one in eight participants was positive for either rectal or pharyngeal chlamydia or gonorrhea. Compared with chlamydia and gonorrhea prevalence estimates among MSM derived from largely clinic-based samples (*5*,*6*), these estimates are lower. Persons screened for STDs in clinical settings (often STD clinics) might represent a population at higher risk (e.g., previous STD, larger number of sexual partners, and known or suspected STD exposure) (*8*). This analysis demonstrates that risk for chlamydia and gonorrhea also might be high among MSM when sampled from nonclinical MSM community venues. This finding suggests that the general population of sexually active MSM might be at elevated risk for STDs and that bacterial STD prevalence estimates from STD clinic-based samples of MSM might not be substantially biased.

The current recommendation for sexually active MSM is to screen for STDs at all exposed anatomic sites at least annually (*4*), and MSM living with HIV infection likely have more opportunities for STD screening when in care. This study found a high prevalence of extragenital chlamydia and gonorrhea among MSM living with HIV, and the findings reinforce current HIV care guidance, which recommends that MSM who report receptive anal and oral sex should be screened for rectal and pharyngeal gonorrhea and chlamydia, respectively, at their initial visit and at least annually thereafter (*9*).

Among this sample of MSM recruited at community venues, approximately one third reported that they had not been tested for an STD in the previous 12 months, suggesting that a substantial number of MSM at high risk for STDs are not being screened per current recommendations. Although the extragenital STD prevalence among these men was high, prevalence was similarly high among MSM who did report having been tested within the past 12 months. Among those tested in the previous 12 months, this survey did not record which testing was performed or how frequently these men were tested. More frequent (e.g., every 3–6 months) screening of MSM with elevated risk might be needed to reduce prevalence among those who are already being screened for STDs.

The findings in this report are subject to at least four limitations. First, NHBS STD screening only included five U.S. cities. Although the cities were geographically and sociodemographically diverse, extrapolation to all U.S. cities is not appropriate. Second, MSM were recruited through community venue–based sampling, not probability sampling; therefore, further extrapolation to the MSM population within the five cities is not possible. Third, this survey was limited to pharyngeal and rectal chlamydia and gonorrhea screening; urogenital or urine specimens were not collected. Most MSM with asymptomatic urogenital infection also are infected at extragenital sites (*10*); therefore, it is unlikely that a large number of infected persons were missed who would have been detected had NHBS conducted urogenital screening. Finally, self-reported data on STD testing in the previous 12 months might overestimate the adherence to current screening recommendations because they included any STD test, not specifically chlamydia and gonorrhea extragenital testing at anatomic sites of exposure.

Among a sample of MSM attending community venues in five U.S. cities, approximately one in eight had an infection with chlamydia or gonorrhea at an extragenital site. According to CDC guidelines, sexually active MSM should be screened at least annually for STDs at exposed anatomic sites, including more frequent screening (e.g., every 3–6 months) in MSM at elevated risk for STDs (*4*,*9*). Despite the screening recommendation, one in three MSM in this study did not report STD testing in the previous 12 months. The asymptomatic nature of extragenital STDs and high prevalences found in this population further support the need for regular screening of all sexually active MSM at all anatomic sites of exposure. Improved access to culturally competent care and clinician adherence to screening guidelines for MSM are critical components in reducing the STD disparities that affect this population.

**Summary**

**What is already known about this topic?**

Men who have sex with men (MSM) are disproportionately affected by sexually transmitted diseases (STDs) and human immunodeficiency virus (HIV) infection. Most MSM STD prevalence data are from STD and HIV clinic attendees.

**What is added by this report?**

Among community venue–attending MSM in five cities in 2017, approximately one in eight had an extragenital chlamydial or gonococcal infection. Rectal gonorrhea prevalence was higher in MSM infected with HIV than in those not infected with HIV.

**What are the implications for public health practice?**

Sexually active MSM should be screened at least annually for chlamydia and gonorrhea at all exposed anatomic sites; some MSM might benefit from more frequent screening.
